# Enhancing Qualities of Consciousness during Online Learning via Multisensory Interactions

**DOI:** 10.3390/bs11050057

**Published:** 2021-04-21

**Authors:** Valentina Cesari, Benedetta Galgani, Angelo Gemignani, Danilo Menicucci

**Affiliations:** 1 Department of Surgical, Medical and Molecular Pathology and Critical Care Medicine, University of Pisa, 56126 Pisa, Italy; valentinacesari91@gmail.com (V.C.); angelo.gemignani@unipi.it (A.G.); 2 Department of Law, University of Pisa, 56126 Pisa, Italy; benedetta.galgani@unipi.it

**Keywords:** education, flow, sense of presence, engagement, social interactions, boredom, multisensory stimulation, virtual reality, augmented reality, mixed reality

## Abstract

Online-learning is a feasible alternative to in-person attendance during COVID-19 pandemic. In this period, information technologies have allowed sharing experiences, but have also highlighted some limitations compared to traditional learning. Learning is strongly supported by some qualities of consciousness such as flow (intended as the optimal state of absorption and engagement activity) and sense of presence (feeling of exerting control, interacting with and getting immersed into real/virtual environments), behavioral, emotional, and cognitive engagement, together with the need for social interaction. During online learning, feelings of disconnection, social isolation, distractions, boredom, and lack of control exert a detrimental effect on the ability to reach the state of flow, the feeling of presence, the feeling of social involvement. Since online environments could prevent the rising of these learning–supporting variables, this article aims at describing the role of flow, presence, engagement, and social interactions during online sessions and at characterizing multisensory stimulations as a driver to cope with these issues. We argue that the use of augmented, mixed, or virtual reality can support the above-mentioned domains, and thus counteract the detrimental effects of physical distance. Such support could be further increased by enhancing multisensory stimulation modalities within augmented and virtual environments.

## 1. Online Learning’s Bedrocks

The widespread use of multimedia and information technologies, as well as the use of the internet, led to a dramatic change in the process of “teaching-learning” [1], and simultaneously generated multiple choices of teaching for higher education. In this vein, institutions such as colleges and universities are now involved in a continuous process with a view to improving online course proficiency [2].

During the COVID 19 outbreak, face-to-face teaching has been subjected to dramatic restrictions for all educational cycles, and online-teaching has become the sole alternative to institutional closure. Students and educators were forced to resort to remote learning for safety reasons without taking into account the several issues of distance learning. Even if online learning has traditionally been adopted as an alternative opportunity especially intended for higher education cycles, the mandatory switch due to the COVID-19 pandemic did not take into account subjective agreement. Indeed, the rapid adaptation to virtual courses posed several issues, such as socioeconomic discrimination, digital gaps, access to learning [3], but also students’ and instructors’ proneness to distance learning modality.

According to the timing of delivery, synchronous online learning is the best alternative to in-person attendance because it takes advantage of real-time sharing sources and communication channels on the internet [4,5,6]. Thus, compared to computer-based and asynchronous online learning, the synchronous online learning modality allows learners to receive immediate feedback [5] and share real-time experience among participants through video conferences, chat rooms, and interactive platforms.

Recent studies have found out that students are likely to experience a relevant degree of psychological distress and negative emotions in online learning modality. In this context, a pivotal role in aggravating psychological distress is played by fragmented perceptions of online learning [7]. Online learning’s inadequate effectiveness might be caused by multiple and heterogeneous factors, such as online course quality, the usability of content, technological ease, access to technical assistance, and degree of interaction with peers during the sessions [8,9]. These factors might foster a “reverberating circuit” in which optimal learning levels could be difficult to reach, or even fully prevented [10].

However, synchronous online learning is the modality that best reproduces face-to-face modality, since it allows restoration of the two following fundamental qualities of conscious experience: flow, that is, the optimal state of absorption and engagement, and sense of presence, that is the feeling of exerting control, interacting with and getting immersed into the real/virtual environment. Both these qualities lead to the “transportation of consciousness into an alternative virtual reality” [11,12,13].

The difficulty in guaranteeing an optimal learning environment might also be due to some difficulties that hinder the rising of flow and sense of presence in human–computer interaction for educational purposes. Moreover, other issues could also negatively affect emotional, behavioral, and cognitive engagement and frustrate the social need for deliberate and spontaneous social interaction and cooperation among users.

When flow, presence, engagement, and interaction among users are weak or even absent, facing online sessions could be hard since higher degrees of self-motivation and time management abilities are required to face the lack of personal relations due to remoteness [14].

Since the ongoing global pandemic constitutes a fertile ground to study physical, cognitive, and emotional barriers to the learning process, this article will describe the role of flow, sense of presence, engagement, and social interactions during online educational sessions. It will try to highlight antecedents, the aftermath of their absence, and drivers to support them. In particular, a possible driver might be to implement online learning settings in multisensory environments.

### 1.1. When “Being Lost in Time and Space” Matters: The Role of Flow in Online Learning

The concept of flow has been introduced by Csikszentmihalyi [15] to identify “an optimal experience” intended as a shift to an experience in which individuals get into an absorption state during a particular activity, while the mind becomes effortlessly focused and engaged. According to Csikszentmihalyi and Csikszentmihalyi [16], being in a state of flow means to be in a state of selected focus of awareness, by which irrelevant elements (i.e., perceptions and thoughts) are excluded to prevent distraction; loss of self-consciousness; high degree of responsiveness to goals and feedback and full management and control exerted toward the surrounding environment.

It is worth noting that flow is not to be intended as an “all-or-nothing” dichotomic state: it is rather a dimensional construct highlighting a continuum from absence to a maximum state of experienced flow.

The state of flow is particularly recurrent in the context of learning, since educational settings have been reported to benefit from the state of flow and vice versa, thus constituting a virtuous circle [17]. However, factors such as a higher degree of experienced boredom and dysfunctional mood monitoring (such as anxiety rumination) could interfere with the state of flow. Boredom, for example, has been found to be an interferential emotion in learning processes, since it increases the perceived time on a task (e.g., time is perceived more slowly [18], and it decreases focused attention [19,20]). Watt [18] reported that individuals with boredom proneness perceived time passing slower during a task, whereas the ability to self-generate stimulation is a factor that could counteract boredom proneness, which in turn, implies difficulty in focusing attention [19,20]. In this line, Aguilera-Hermida’s study [21] reported that mandatory shift from face-to-face to online learning due to COVID-19 outbreak produced several detrimental effects, with a decrease in motivation, self-efficacy, and cognitive engagement during learning sessions.

On the contrary, other studies have suggested that some factors could be elicited by particular crisis and emergence conditions (such as this unprecedented pandemic time) and might lead people to a copying process by enhancing flow state and learning opportunities [22,23]. For example, in the recent study conducted by Bawa [24], which aimed at comparing the proficiency achieved by students who attended online courses during the COVID-19 outbreak and students who attended face-to-face classes before the pandemic emergency, the online classes achieved better academic results. Bawa [24] suggested that their better performance could have been associated with the enhancement of flow state.

This assumption is in line with the VUCA model, which summarizes the factors favoring learning performances; indeed, VUCA is an acronym constituted by its four main factors: “Volatility (rapidly changing contexts and conditions), Uncertainty (information missing that is critical to problem-solving), Complexity (multiple factors difficult to categorize or control), and Ambiguity (vague data subject to multiple interpretation)” [25], p 16. In sum, the acceptance of online learning sessions seems to be the conditio sine qua non for a flow experience, which, in turn, offers a primary support in the learning process via cognitive, emotional, and dispositional variables.

### 1.2. Feeling There, Being Here: The Role of Presence in Online Environment 

Presence is intimately linked to the body and to the embodiment process [26,27,28,29]. As claimed by Biocca [26], “before paper, wires, and silicon, the primordial communication medium is the body. At the center of all communication rests the body, the fleshy gateway to the mind. Thinking of the body as an information channel, a display device, or a communication device, we emerge with the metaphor of the body as a kind of simulator for the mind.” Following Biocca [26] and the primary role of the body in supporting the sense of presence, it seems utterly impossible to re-think the online-learning environment as a scenario allowing to achieve the sense of presence. As distance learning continues to expand in order to cater to larger numbers of students across the globe, current online learning modalities provide inefficient education delivery systems since they are not immersive, sufficiently engaging, and motivating, thus resulting in poor rates of completion [25,30,31,32]. The term “telepresence” had been coined by Minsky [33] to depict the phenomenon of “being there” by users of teleoperation systems in a virtual environment. Following Steuer [34], telepresence is determined by a high degree of interactivity and vividness.

In order to create the sense of presence in the online environment, a restructuring of thought, feeling, and behavior is necessary: in fact, as claimed by Biocca et al. [27] “we have to make an effort to be aware of the intentions of others and their thoughts, emotions, and behaviors when they are connected to us via technology”. The higher experience of telepresence in online sessions could allow users to interact with the learning activities themselves deeply. Most importantly, the creation of telepresence in the online environment was identified to play a pivotal role in creating the state of flow: indeed, this variable has been included in various studies to show that when users perceive a sense of telepresence in mediated (i.e., not physical) environments, this can create a perceptual illusion of being present and highly engaged, thus allowing flow to occur [26,35]. In order to investigate the role of telepresence in determining flow, Guo et al. [36] empirically tested a model that takes into account several factors in promoting users’ flow in online learning and users’ online continuance intentions. Among these factors, telepresence resulted in being the most influencing variable in concurring to flow experience.

### 1.3. Virtually (Dis)Connected: The Importance of Social Interaction in Online Environments

Merleau-Ponty’s philosophy stated that communicators can fully experience the alterity of the other only in concrete, temporal, and spatial situations, i.e., in face-to-face encounters where direct perception can take place (Merleau-Ponty, cited in [37], p. 403). Physical separation, lack of interaction with educators and peers [38], and lack of social cues are urgent issues in attending online courses since they represent a risk of experiencing feelings of isolation [39,40]. Moreover, conventional socialization that normally occurs in face-to-face learning, is missing in online modality due to the poor quality of real-time sharing of ideas, knowledge, and information [41]. Physical separation reduces users’ sense of community which, in turn, intensifies feelings of disconnection, isolation, distractions, and lack of attention [39]. Importantly, the lack of social interaction was the most severe barrier perceived by students during online learning, as reported by Muilenburg and Berge [40]. Impaired social interactions might induce a lower degree of users’ satisfaction, higher feelings of disillusionment, and most importantly, it constitutes a tangible risk of dropping out of online courses [42,43,44,45]. Lack of sociability during online sessions, diminished group cohesion, and the instability of social space might lead to decreased social presence, that is, an altered “perceived degree of illusion that the other in the communication appears to be a real physical person in either an immediate (i.e., real time or synchronous) or a delayed (i.e., time-deferred or asynchronous) communication episode” [46], p 185. The improvement of social interactions during online sessions might constitute an imperative for a more effective and enjoyable educational experience.

Lack of socialization and interaction are central themes of the COVID-19 pandemic restrictions, in which physical distance was unavoidable to minimize the virus spread. In this context, Adnan and Anwar [47] found that students perceived the lack of face-to-face interactions with the educator, together with the absence of conventional socialization between peers and educators, as urgent issues to be solved. In fact, the authors reported that 42.9% of students complained about difficulties in doing group projects due to a lack of socialization during online learning modality. Similarly, Nambiar’s work [48] showed that 15.4% of students reported that online classes were less interactive and less communicative. Other surveys, which aimed at investigating the impact of online learning on students worldwide, found that social interactions constitute a crucial issue to deal with to improve the learning process [49,50,51]. Interestingly, the study performed by Baber [52] showed a peculiar phenomenon during the COVID-19 lockdown in which VUCA factors acted to foster the qualities of consciousness without requiring the motivational support linked to social factors. Results highlighted the primary importance given to social interaction in improving online learning by students attending online courses, but its effect was reduced when the social distance was prescribed, as people give more importance to containing the COVID-19 spread rather than socializing in the online environment.

In summary, the role of social interactions during online learning constitutes a core variable to achieve optimal learning outcomes, and it does not play a marginal role in this complex process. In fact, the virtual social disconnection could prompt negative states (feelings of disconnection, isolation, distractions, and lack of attention) whose outcomes could lead to less effective learning, lower performance, and even drop out of the courses.

### 1.4. (Not) Bored Out of Mind: The Importance of Deep Engagement in Online Settings to “Fit in”

Within the educational field, several studies have focused on the set of emotions, such as enjoyment and boredom, that are thought to predict learning skills, self-regulatory capacity, and educational goals among students [53,54,55,56]. The experience of boredom includes attentional decrement, negative affect, non-optimal physiological arousal (low or high arousal), and difficulty in concentrating: all these factors might prevent real and deep subjective engagement in the learning process [57,58]. According to contextual theories of boredom, lack of stimulation could prompt a state boredom, which exerts a detrimental effect on learning skills. From a cognitive perspective, it has been suggested that boredom arises through a three-stage model: (1) subjective difficulty in being engaged—in terms of attention—in a satisfying activity, (2) awareness of lack of engagement, and (3) the attribution of lack of engagement to the nature of the activity itself [59].

As regards the relationship between boredom and attentional deficiencies, some studies reported that poor attentional engagement and attentional failures during task completion might lead to a state of boredom [60,61]. These results, thus, suggested that disengagement—reflected in inattention—precedes feelings of boredom.

Some studies have highlighted factors such as lack of stimulation, technical issues, social isolation, and lack of spontaneous interaction among students that could lead to boredom in online settings, and have pointed out the role of boredom in preventing a high degree of self-regulation and the adequate engagement required in online learning [14].

Artino and Jones II’s work [62] showed that subjects who experienced a high degree of boredom during online courses were likely to have less adaptive strategies, indicated by a lower degree of metacognition and elaboration.

The study carried out by You and Kang [63] highlighted that higher experienced boredom during online courses displayed, together with anxiety, a moderating effect on the subjective ability of self-regulating strategies. It is worth noting that self-regulation strategies represent core factors in learning, and they could be defined as the level of subjective abilities in the learning process using metacognition and motivation [64].

The recent study by Martarelli et al. [65] investigated the role of the relationship between self-control, boredom, and online learning performance during COVID-19 pandemic. They found that students with high self-control perceived online courses as less difficult, and their perceptions improved students’ adherence to online sessions; conversely, students more prone to experience boredom perceived online courses as more difficult, and this negatively affected their adherence to online sessions.

## 2. Online-Learning Enhancement: Multisensory Training to Face Online Learning Issues

*“If the virtual reality apparatus, as you called it, was wired to all of your senses and controlled them completely, would you be able to tell the difference between the virtual world and the real world? What is real? How do you define real? If you’re talking about your senses, what you feel, taste, smell, or see, then all you’re talking about are electrical signals interpreted by your brain.”*

From *‘The Matrix’* movie, 1999

Everyday experiences often include simultaneous stimulations of multiple sensory modalities [66]. A paradigmatic example of this multisensorial stimulation is offered by the cooking experience, as claimed by Ferran Adrià, “Cooking is the most multi-sensual art. I try to stimulate all the senses” [67]. In this context, Spence et al. [67] distinguished sensory cues into the following two categories: those belonging to flavor (namely, retronasal olfaction, gustation, oral-somatosensory, and trigeminal inputs), and those belonging to food-related sensory cues (such as visual, auditory, and orthonasal olfactory cues), that promote the generation of flavor expectations. This digression helps us to understand the way we are subjected to this “storm of the senses”. Imagine the following scene: a cooking trainer tries to teach a recipe to their student from their personal computer. Could a student imitate what they are doing by using visual and auditory senses? What about the final product? How could the trainer effectively verify the final product without smelling or touching it? These issues should be taken into account when an online learning course is delivered and, most importantly, they should be not restricted to hands-on, practical skills, such as cooking—in which a high degree of ineffectiveness of online-learning has been pointed out [68]. In the educational field, learning enhancement through multisensory stimulation has clearly been established. The multisensory modality has been strongly supported by the educational pioneer Maria Montessori [69] for more than 100 years; she encouraged learning through the simultaneous stimulation of the senses using a mixture of visual, tactile, and kinesthetic method: for example, teaching, writing, and reading are not limited to the visual approach, but they also involve tactile and auditory senses; similarly, maths can be deeply understood via tactile manipulation. It has been found that students reach higher scores on academic tasks with the Montessorian approach [53,70,71]. Moreover, the multisensory learning approach has the advantage of engaging people who developed different sensory modality to retain learning contents: for example, some people are mostly visual, whereas others are mostly auditory. Despite the “sensory individual preference” during learning processes, the combination of multiple senses stimulation augments the retention of learning-related contents [72].

The multisensory stimulation can occur both in a real environment, as stated with the Montessorian approach, and in virtual, augmented, or mixed reality environments. Virtual reality (VR), augmented reality (AR), or mixed reality (MR) technologies typically alter perception [73]. These technologies generally target visual and auditory perceptions, although haptic (touch), smell (olfaction), or taste (gustatory) stimulation systems have also been developed [66,74]. As regards differences between VR, AR, MR, VR technologies create an artificial environment allowing the user to interact with it at the expense of the abilities to interact with the real world deeply; differently, AR and MR technologies enrich real environment perceptions. VR simulations provide valid alternatives for applications in training, education, research, entertainment, and clinical fields as they promote the subjects’ immersion in and interaction with near-realistic 3D scenarios [75,76,77]. In this way, VR enhances the subjective sense of presence by the constant interaction and manipulation of the virtual scenario [78]. At present, VR systems show an evolution towards the use of multisensory stimulation in order to enhance the sense of presence: indeed, a strong empirical background indicates that the more senses are engaged, the more immersive the user’s experience and the better proficiency will be achieved [66]. However, this development is still embryonic if compared to AR applications. The AR context Hashimoto et al. [79] implemented the Straw-user Interface to elicit drinking experiences using pre-recorded data of sounds, pressures, and vibrations. Ikeno and colleagues [80] implemented AR saki bottles to study the effects of audio-haptic feedback on the drinking experience. Another example came from “Vocktail”, an AR tool augmenting flavor experiences via a mixture of taste, smell, and visual stimuli, implemented by Ranasinghe et al. [81] that includes Digital Lollipop, Virtual Lemonade, FunRasa, and Taste, all of which using electrical and thermal stimulation over the tongue to simulate sour, salty, bitter, and sweet tastes.

Moreover, in the AR context, holography (conceived in 1947 by Dennis Gabor [82]) is a central theme: based on this technique, holographic telepresence can be created for enhancing engagement in the learning process.

### 2.1. Stimulating Flow during Online Session Using AR/VR Systems to Enhance Learning

The widespread use of AR/VR in the educational field has allowed investigation of their effects on the key features of flow, such as state of engagement, intrinsic motivation, proneness to retain learning material, absorption, time distortions, etc. In this context, following the literature review conducted by Chen et al. [83], AR devices have been found to promote learning gains and motivation, engagement, and positive emotional states such as enjoyment. For example, Ibáñez et al. [84] compared the degree of experienced flow during the teaching of electromagnetism principles in students using the AR learning modality and students using a web-based application. Results have shown that in the AR group, a high number of features contributing to flow enhancement have been experienced: concentration, altered time perception, sense of control, clearer direct feedback, and autotelic experience. Moreover, the authors suggested that the aforementioned features may enhance flow state by promoting specific learners’ psychological states, such as the construction of identity, sense of presence, and co-presence [84]. Similarly, Tuli et al. [85] found that AR technologies help students to reach a higher level of flow and better educational proficiency during electromagnetic principles lessons. Within the field of informatics, it has been shown that AR improves learning contents retention and supports the experience of flow, which, in turn, helps to improve students’ performance [86]. Authors suggested that AR “engenders impression and interest to students, which has, as a result, to motivate them more, to participate more actively and with more enthusiasm in course activities, to be more concentrated and comprehend better anything they are taught” [86], p. 86. Regarding VR devices, a meta-analysis evidenced that games, simulations (imitation of real-life actions), and virtual worlds (creation and manipulations of objects in virtual environments) provide improvements in learning outcomes; more specifically, games show higher learning gains than simulations and virtual worlds [87]. Moreover, the recent work by Wang et al. [88] analyzed the answers given to online questionnaires by a total of 296 students using the structural equation model: results reported the primary role of flow and perceived value for users during online sessions.

### 2.2. Supporting Presence during Online Session Using AR/VR Devices to Enhance Learning

The “bedrock” of VR dwells in the user’s immersive experience of virtual scenarios: this immersion could foster the transfers of learned skills from VR to real-world contexts, thanks to its ecological validity [89]. Presence is suggested to be the mediator by which many VR applications achieve their goal [90]. However, even the lower degree of immersions offered by AR devices, delivered in full 3D at 30 frames per second (or faster), combined with shared AR workspaces, could dramatically reduce the distance between users [91,92,93]. Promising results in augmenting the sense of presence derive from holographic technology, whose adoption is increasing in medical education, where the relation between the enhancement of the sense of presence and the improvement in the learning process has been highlighted. In this context, Orcos and Magreñán [94] found that holographic devices improved, compared to classical teaching, the acquisition of biological contents, and augmented student satisfaction and motivation towards holographic learning-content.

Other beneficial effects of holography on learning outcomes come from the study conducted in the medical education field [95,96]. The recent study by Li and Lefevre [97] evidenced the effectiveness of holographic devices using avatars in enhancing the sense of presence during online seminars. Results indicated that this modality intensifies the teaching presence of remote speakers, the engagement between participants and the enjoyment of seminar/lessons attendance.

### 2.3. Sustaining “Near-Realistic” Interactions during Online Sessions Using Multi-Users Functionality

One of the advantages of VR/AR is the possibility of cooperative virtual sessions using multi-user functionality; the simultaneous interaction between users. The multi-user virtual environment allows access, interacts with, and manipulates digital content, representing people in virtual scenarios through virtual human-like figures (i.e., avatars), communicating with other participants, and taking part in experiences resembling real-world interactions [98]. In this way, participants in different physical locations could reach more immersive and realistic interactions by exploring and manipulating the same virtual environment. With multiple users, VR devices could promote collaborative learning processes where students learn together and often from each other [99]. Collaborative learning strategies play a role in increasing student performance, interpersonal (group cohesion), and personal skills (self-esteem, high-level thinking) [100,101]. Creating a collaborative virtual environment could foster individual motivation, which in turn, could help students to reach higher academic results. The creation of a collaborative virtual classroom is believed to increase motivation and performance among students. For example, Monanan et al. [102] devised a Collaborative Learning Environment with Virtual Reality (CLEV-R), in which participants can learn content, collaborate during sessions, and even socialize during recreational moments. With a view to promoting genuine socialization, authors implemented a virtual common area with round tables; these tables were in a “coffee area” and were used to engage in conversations with others, thus guaranteeing the satisfaction of social needs. Similarly, the investigation performed by Edirisingha et al. [103] pointed out the importance of satisfying social needs during archeological lessons using students’ social engagement. Another beneficial effect of collaborative VR online modality on learning processes comes from Lorenzo et al. [104], who reported that the use of the Massively Multi-User On-line Learning (MMOL) Platform setting allowed users to reach more interpersonal interactions and genuine participation during learning tasks, thus suggesting enhancement of group relationships.

### 2.4. Counteracting Boredom with Stimulating Engaging Scenarios

One of the most fundamental properties of VR/AR devices is the ability to “engage” subjects [105] by promoting multiple and personalized learning methods [106]. In this way, AR/VR tools could minimize the emergence of state boredom by using different types of stimulations and by enriching learning environments. For example, the study conducted by Allcoat and von Mühlenen [107] reported that students using VR tools, as compared to those using traditional and video learning methods, reported higher engagement and more positive academic grades. A smart way to contrast boredom was proposed in Patel’s work [108] by introducing virtual “escape rooms”. Thanks to escape rooms, subjects had the opportunity to exit the classroom for a pause and to come back later. Patel’s work [108] has shown that the usage of escape rooms promoted engagement, counteracted boredom, and enhanced learning during the COVID-19 pandemic. Moreover, special games based on knowledge gained from course content were implemented in order to allow the subject’s exit via escape rooms, thus motivating subjects to retain and apply learned information.

## 3. Conclusions

**Figure 1:**
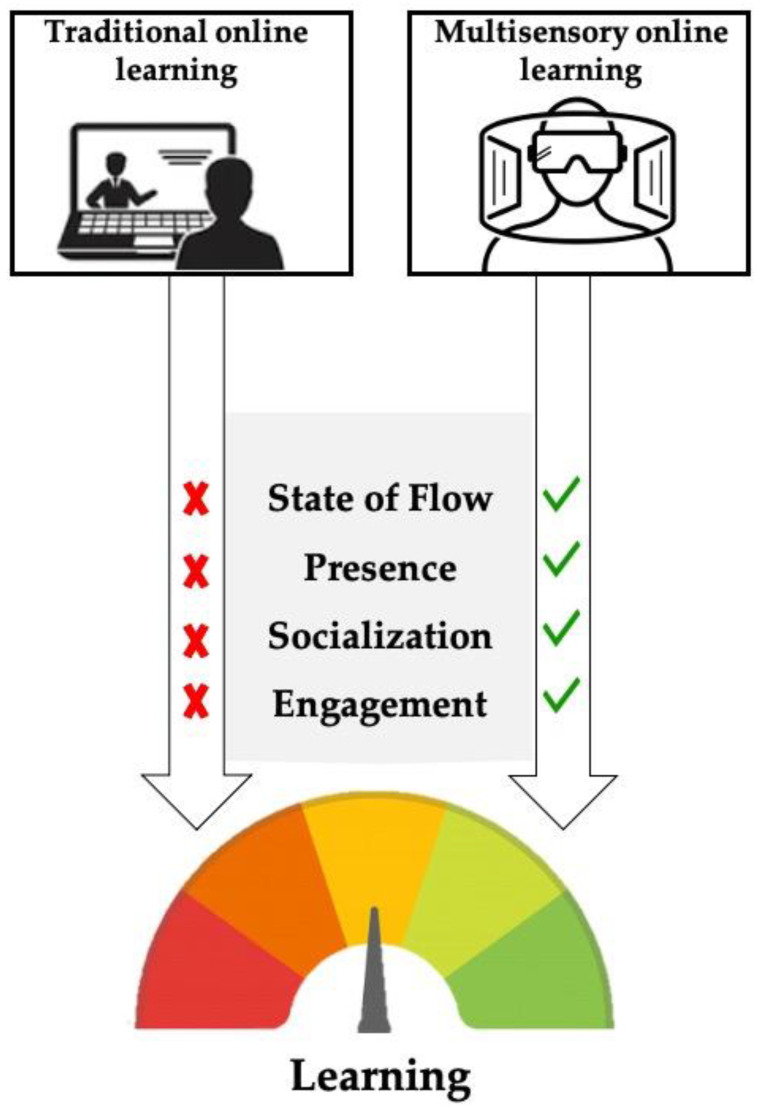
Schematic flow chart of the online learning process. On the left: traditional online learning prevents the reaching of learning bedrocks, thus leading to learning impairment. On the right: multisensory online learning allows the reaching of learning bedrocks, thus leading to learning empowerment.

Learning is a complex process that cannot be reduced to a mere cognitive factor. This unprecedented pandemic has highlighted the importance of face-to-face interactions during the process of “teaching-learning”. During online learning, the ability to reach an optimal state of flow, the feeling of presence by which we exert control over the environment we are immersed into, and the ability to be engaged within the online classes in order to feel part of a peer community during learning sessions or even enjoy recreational moments are highly interdependent aspects and-as such-strongly support the online learning process. However, it is fairly easy to fall into a reverberating vicious circle when attending online lessons, since the lack of flow state, sense of presence, socialization, and engagement exerts a detrimental effect on learning abilities (Figure 1, left). For this reason, the use of online learning should imply a total restructuring process to facilitate users’ experience. Drawing inspiration from the strong background on the effectiveness of multisensory stimulation to enhance learning, several devices have been implemented to facilitate a “near-realistic” experience, allowing the diffusion of AR/VR devices to support this complex process. A case in point is the enhancement of social presence by using avatars (also with the help of holographic technology), or other tools: although they do not fully replace face-to face-interactions, they might alleviate users from negative effects by enhancing flow state, sense of presence, socialization, and engagement, thus guaranteeing an adequate learning experience (Figure 1, right).

Finally, it is worth stressing that all suggested interventions require appropriate infrastructural conditions currently not available everywhere. Indeed, according to UNESCO data [109], less than half the worldwide population has access to the internet. The digital divide, that is “the patterns of unequal access to information technology based on income, race, ethnicity, gender, age, and geography” [110], has made it difficult for some students to move from face-to-face to online learning during the COVID-19 pandemic and this difficulty has led to more evident social inequalities among students, compared to traditional (i.e., face-to-face) learning modality.

Another important issue that has to be taken into account is the age-related digital divide between teachers and students in terms of technology, since online learning requires a dual activity to be performed. The abrupt switch caused by COVID-19 has forced educators to online learning regardless of their technological skills. Consequently, teachers have faced and are still facing significant challenges in adapting to online teaching, and maintaining an adequate level of communicative skills with students, and in supporting students’ process of learning.

Despite these limitations, we suggest multisensory stimulation in VR/AR environment to be a powerful tool to mitigate issues related to distance learning modality.
